# Maintenance of response with atypical antipsychotics in the treatment of schizophrenia: a post-hoc analysis of 5 double-blind, randomized clinical trials

**DOI:** 10.1186/1471-244X-9-13

**Published:** 2009-03-31

**Authors:** Virginia Stauffer, Haya Ascher-Svanum, Lin Liu, Tamara Ball, Robert Conley

**Affiliations:** 1 Lilly Research Laboratories, Indianapolis, IN, USA; 2 i3 Statprobe, Ann Arbor, MI, USA

## Abstract

**Background:**

How long an antipsychotic is effective in maintaining response is important in choosing the correct treatment for people with schizophrenia. This post-hoc analysis describes maintenance of response over 24 or 28 weeks in people treated for schizophrenia with olanzapine, risperidone, quetiapine, ziprasidone, or aripiprazole.

**Methods:**

This was a post-hoc analysis using data from 5 double-blind, randomized, comparative trials of 24 or 28 weeks duration in which olanzapine was compared to risperidone (1 study; N = 339), quetiapine (1 study; N = 346), ziprasidone (2 studies; N = 548 and 394) or aripiprazole (1 study; N = 566) for treatment of schizophrenia. For each study, time to loss of response in patients who met criteria for response at Week 8 and the proportion of patients who lost response following Week 8 were compared by treatment group. The number needed to treat (NNT) with olanzapine rather than comparator to avoid loss of one additional responder over 24 or 28 weeks of treatment was calculated for each study.

**Results:**

Time maintained in response was significantly longer (p < .05) for olanzapine compared to risperidone, quetiapine, and ziprasidone. Olanzapine did not significantly differ from aripiprazole. The proportion of patients who lost response was significantly lower for olanzapine versus risperidone, quetiapine, and ziprasidone (p < .05). NNTs to avoid one additional patient with loss of response with olanzapine versus risperidone, quetiapine and ziprasidone were favourable, ranging from 5 to 9.

**Conclusion:**

During 24 and 28 weeks of treatment, the antipsychotics studied differed in the time that treated patients with schizophrenia remained in response and the proportion of patients who lost response. Olanzapine treatment resulted in a consistent and statistically significant advantage in maintenance of response compared to treatment with risperidone, quetiapine and ziprasidone; but not compared to treatment with aripiprazole.

## Background

The characteristics of response to antipsychotic medication in the treatment of schizophrenia are an important determinant of adherence to treatment and a predictor of long-term functional outcome [1,2]. In multiple large, randomized, double-blind studies of antipsychotic efficacy, patients identified lack of efficacy more commonly than medication intolerance as the reason they discontinued treatment, and patients' subjective assessment of lack of efficacy was corroborated by objective measures of psychopathology [3,4]. In addition, for patients who initially experienced response but later discontinue treatment, discontinuation was frequently preceded by symptom worsening [5].

In a large meta-analysis in which efficacy was primarily measured as a change from baseline in Positive and Negative Syndrome Scale [6] (PANSS) Total score or Brief Psychiatric Rating Scale [7] (BPRS) score, significant differences were seen between first and second generation antipsychotics and between individual second generation agents [8]. However, as noted by Leucht et al. [9], these symptom rating scales are not familiar to or commonly used by practicing clinicians, and categorical definitions of "response" and "nonresponse" based on valid scale-derived cut-offs may offer more clinical usefulness. Leucht et al. recently correlated PANSS Total scores to scores on the Clinical Global Impression Scale [10] (CGI), an anchored, single dimensional impression of a patient's overall clinical severity. This allowed for specific percentages of improvement over baseline on the PANSS score to be linked to categories of minimal, moderate, and much improvement [11]. However, in the literature, there has been widespread use of different thresholds to define response, and concern has been raised that study results might differ substantially depending on which threshold was chosen [12].

Beyond response, clinicians, patients, and families are interested in sustained response, remission, relapse, and recovery [13]. These constructs demand new ways of evaluating treatment efficacy – ways that include both a measure of symptom severity and a time component. The Clinical Antipsychotic Trials of Intervention Effectiveness (CATIE), a large, randomized, double-blind, 18-month National Institutes of Mental Health-sponsored trial, included three outcome measures that incorporated both time and severity: time to discontinuation due to lack of efficacy; PANSS Total scores and CGI scores over time; and time spent in successful treatment, where "successful treatment" was defined using CGI score-based thresholds [3].

In this analysis, we assess cumulative time spent in response and time maintaining response, defining response by changes in CGI, a global measure of illness severity, and the more symptom-based PANSS Total score. We use data from five long-term, randomized studies in which olanzapine was compared to another atypical antipsychotic. The objectives for each study individually are to compare by treatment: time maintaining response, proportion of patients losing response, number needed to treat (NNT) with olanzapine rather than comparator to prevent one additional loss of response, and cumulative days spent in response.

## Methods

The following criteria for study inclusion were determined *a priori*: 1) randomized, double-blind, and active-controlled trial of olanzapine versus at least one other atypical antipsychotic; 2) duration of 24 to 28 weeks; 3) efficacy assessed using the PANSS and the CGI – Severity Index (CGI-S); 4) participants with schizophrenia, schizophreniform disorder, or schizoaffective disorder (DSM-IV-TR criteria); and, 5) original dataset available to authors.

Five studies, all from within the Eli Lilly and Company Clinical Trial Database, met inclusion criteria, including 1 trial each comparing olanzapine to risperidone [13], quetiapine [14], and aripiprazole [15], and 2 trials comparing olanzapine to ziprasidone [16,17]. Studies were carried out at multiple sites, either internationally [13,15,17] or within the United States [14,16]. Three of the 5 studies enrolled patients with high levels of baseline illness severity (group mean PANSS Total range: 95–102) [13,15,17]. The remaining 2 studies enrolled patients selected for specific characteristics, and these patients tended to be less ill at baseline (group mean PANSS Total range: 79–85). One study included evaluation of patients with prominent depressive symptoms [16], and the other enrolled patients with prominent negative symptoms and poor functioning [14]. The 5 studies are summarized in Table 1, and detailed descriptions are available in their respective published reports [13-17].

**Table 1:** Characteristics of the 5 source studies used in these analyses.

**Primary ** **reference**	**Primary ** **outcomes**	**Study ** **drugs**	**N**	**Mean** **modal** ** dose** **(mg/day ** **[SD])**	**Study ** **duration** **(weeks)**	**Diagnoses**	**Other ** **baseline ** **inclusion** **criteria**
Tran [13]	Efficacy Safety	Olanzapine Risperidone	172 167	17.2 (3.6) 7.2 (2.7)	28	Schz, Schzfm, Schzaff	Inpatient and outpatient Age 18 to 65 BPRS (ext) score ≥ 42

Kinon [14]	Negative Symptoms Functional Outcome Efficacy Safety	Olanzapine Quetiapine	171 175	15.6 (4.3) 455.8 (156.3)	24	Schz, Schzaff	Outpatients Age 18 to 65 Score ≥ 4 on at least 3, or ≥ 5 on at least 2 of the 7 negative symptom items of the PANSS, and ≥ 60 (moderate difficulties) on the GAF.

Breier [17]	Efficacy Safety	Olanzapine Ziprasidone	277 271	15.3 (4.5) 116.0 (39.9)	28	Schz	Inpatient and outpatient Age 18 to 75 Scores ≥ 42 on the BPRS (ext), ≥ 4 on at least one positive symptom item of the PANSS, and ≥ 4 on the severity of illness subscale of the CGI

Kinon [16]	Depressive Symptoms Efficacy Safety	Olanzapine Ziprasidone	202 192	14.2^a^ 110.2^a^	24	Schz, Schzaff	Inpatient and Outpatient Age 18 to 60 Scores ≥ 16 (mild depression) on the MADRS and ≥ 4 (pervasive feelings of sadness or gloominess) on item 2 (reported sadness) of the MADRS

Kane [15]	Efficacy Safety	Olanzapine Aripiprazole	281 285	16.7 (2.4) 19.3 (6.8)	28	Schz	Initial PANSS Total score of ≥ 75, a minimum score of ≥ 4 on one of the PANSS positive, and a minimum score of 4 on the CGI-S at both visits 1 (screening) and 2 (randomization), with an initial score of ≥ 3 on the CGI-I at visit 2.

Abbreviations: Abbreviations: Schz = Schizophrenia; Schzfm = Schizophreniform Disorder; Schzaff = Schizoaffective Disorder; N = number; NNTs = numbers needed to treat; NNHs = numbers needed to harm; BPRS (ext) = Brief Psychiatric Rating Scale (scored 0–6) extracted from the Positive and Negative Syndrome Scale [7]; PANSS = Positive and Negative Syndrome Scale (scored 1–7) [6]; CGI = Clinical Global Impression Scale [10]; MADRS = Montgomery-Asberg Depression Rating Scale; GAF = Global Assessment of Functioning Scale; SD = standard deviation.

^a ^This study had multiple fixed doses, and therefore, SD is not given.

Antipsychotics were dosed within a specified range at clinician discretion, except in one study in which multiple fixed-dose design was used [16]. A limited number of concomitant psychotropic medications were permitted: benzodiazepines/hypnotics; anti-Parkinson medications (for treatment of, but not for prevention of extrapyramidal symptoms); and, in two studies [14,16], fixed doses of antidepressants if the patient had used them in the 30 days prior to enrollment.

For all studies, efficacy and safety outcomes were assessed at intervals of no greater than 4 weeks. When patients discontinued treatment prior to study end, investigators were required to record the date of discontinuation and to complete a checklist of potential reasons for discontinuation.

A total of 2,193 men and women aged 18 to 70 years were randomized to treatment. All protocols were approved by the ethical review boards responsible for individual study sites and all patients or their legal guardians provided written, informed consent consistent with the Helsinki declaration prior to receiving any study therapy or undergoing any study procedure.

### Definitions

Clinical response was defined as a ≥ 20% improvement over baseline PANSS_1–7 _Total score ("minimal clinical improvement." [11]). This threshold has been widely used in antipsychotic efficacy studies and allowed for extension of similar work already reported that used a smaller number of studies for analysis [18]. Loss of response was defined as a ≥ 20% worsening of PANSS_1–7 _Total score and a CGI-S score ≥ 3 occurring any time after Week 8 in a patient who had met response criteria at Week 8. Use of PANSS and CGI-S scores allowed for both an objective, symptom-based evaluation and a more global, clinical evaluation of response. Week 8 was chosen because although many patients do respond quickly (i.e. within the first 2 weeks), there is a subset of patients who will not respond for up to 8 weeks [19]. Waiting 8 weeks ensured that most responders were included, and was consistent with current schizophrenia treatment guidelines, which recommend waiting up to 8 weeks for a response before changing to a different antipsychotic [20,21].

### Statistical Analysis

All of the analyses were completed for each of the five studies individually, and tests of hypotheses were performed at a two-sided significance level of .05. As was done in each of the 5 source studies, the 30 PANSS items were scored from 1 (symptom not present) to 7 (symptoms extremely severe), and PANSS_1–7 _Total scores ranged from 30 to 210.

Treatment differences by therapy group in time to loss of response in patients who met criteria for response at Week 8 were estimated using the Kaplan-Meier technique and compared using the log-rank test. Study endpoints were defined as 196 days for 28-week studies, and 168 days for studies lasting 24 weeks. Data gathered beyond established endpoints were not considered in these analyses.

As a sensitivity analysis, all calculations were repeated with response defined as a ≥ 30% reduction from baseline PANSS Total score, and with the PANSS scored by an alternative system, the "corrected PANSS," or PANSS_0–6_. In this system, each of the 30 items was scored from 0 to 6 rather than 1 to 7, and Total scores ranged from 0 to 180 [9]. Also, in the sensitivity analysis, loss of response was defined as a ≥ 30% worsening of PANSS_0–6 _and a CGI-S score ≥ 3 anytime after Week 8 in patients who met response criteria at Week 8.

Between-group differences in the proportion of patients who lost response after Week 8 after having met criteria for response at Week 8 were assessed using Fisher's exact test. To provide a clinical context for these results, the number needed to treat (NNT) with olanzapine rather than comparator to avoid loss of one additional responder over 24 or 28 weeks of treatment was calculated for each study. NNT was calculated as 1/Absolute Risk Reduction, with 95% Confidence Interval (CI) calculated as previously described [22]. By convention, positive numbers for NNT favoured olanzapine, and negative numbers favoured the comparator. Confidence intervals that included both a positive and a negative number indicated no significant difference between treatments.

Treatment-specific differences in the proportion of time spent in response were calculated for each treatment group using data from patients who had at least one post-baseline PANSS score. Cumulative days spent in response were estimated as follows: if a patient met response criteria at two consecutive visits, all days between visits were tallied; if a patient met response criteria at one of two consecutive visits, 50% of the days between visits were tallied. The proportion of days spent in response was calculated by dividing the cumulative days spent in response by the length of the study. Between-group differences for percentage of days spent in response as a measure of cumulative time spent in response were assessed by the Wilcoxon rank sum test.

## Results

**Figure 1:**
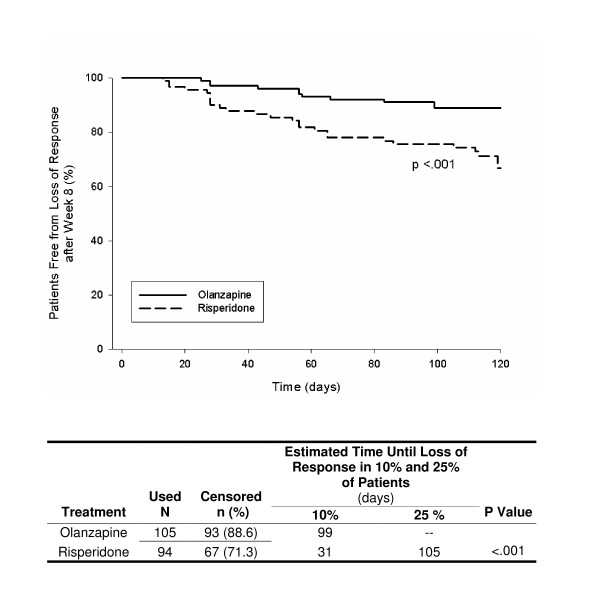
**Kaplan Meier (KM) Analysis of olanzapine versus risperidone for days to loss of response, where loss of response was defined as a ≥ 20% worsening of PANSS_1–7 _Total score and a CGI-S score ≥ 3 in patients who had a ≥ 20% improvement over baseline PANSS_1–7 _Total score at Week 8**. Olanzapine-treated patients remained in response for significantly longer than patients treated with risperidonse (p < .001).

**Figure 2:**
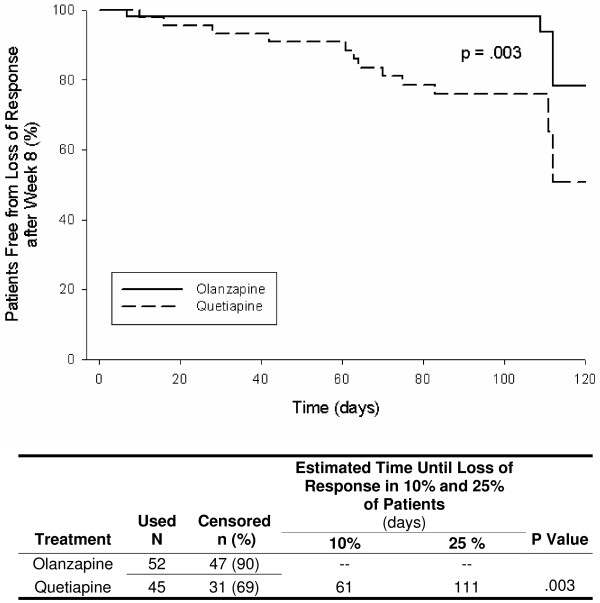
**Kaplan Meier (KM) Analysis of olanzapine versus quetiapine for days to loss of response, where loss of response was defined as a ≥ 20% worsening of PANSS_1–7 _Total score and a CGI-S score ≥ 3 in patients who had a ≥ 20% improvement over baseline PANSS_1–7 _Total score at Week 8**. Olanzapine-treated patients remained in response for significantly longer than patients treated with quetiapine (p = .003).

**Figure 3:**
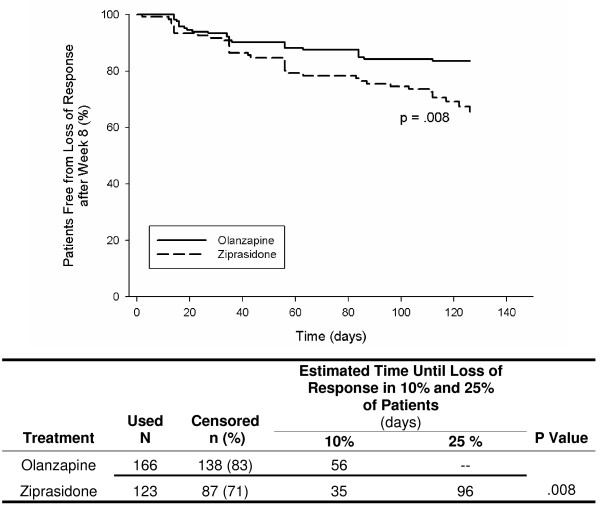
**Kaplan Meier (KM) Analysis of olanzapine versus ziprasidone for days to loss of response, where loss of response was defined as a ≥ 20% worsening of PANSS_1–7 _Total score and a CGI-S score ≥ 3 in patients who had a ≥ 20% improvement over baseline PANSS_1–7 _Total score at Week 8**. Olanzapine-treated patients remained in response for significantly longer than patients treated with ziprasidone (p < .008).

**Figure 4:**
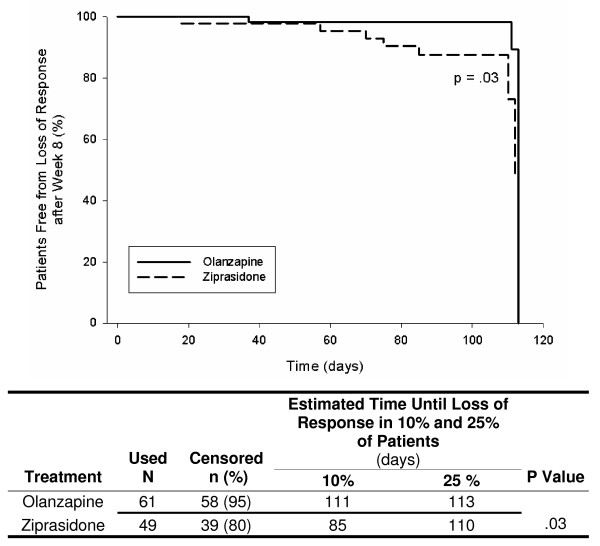
**Kaplan Meier (KM) Analysis of olanzapine versus ziprasidone for days to loss of response, where loss of response was defined as a ≥ 20% worsening of PANSS_1–7 _Total score and a CGI-S score ≥ 3 in patients who had a ≥ 20% improvement over baseline PANSS_1–7 _Total score at Week 8**. Olanzapine-treated patients remained in response for significantly longer than patients treated with ziprasidone (p = .08).

**Figure 5:**
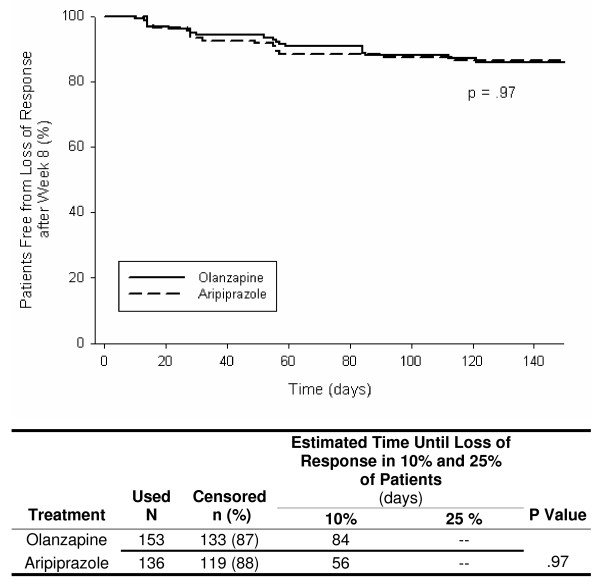
**Kaplan Meier (KM) Analysis of olanzapine versus aripiprazole for days to loss of response, where loss of response was defined as a ≥ 20% worsening of PANSS_1–7 _Total score and a CGI-S score ≥ 3 in patients who had a ≥ 20% improvement over baseline PANSS_1–7 _Total score at Week 8**. There was no significant difference between treatment groups for time remaining in aripiprazole (p = .97).

Figures 1, 2, 3, 4, 5 show results of the KM analyses of olanzapine versus comparator for time to loss of response, where loss of response was defined as a ≥ 20% worsening of the PANSS_1–7 _Total score and a CGI-S score ≥ 3 in patients with a ≥ 20% improvement over baseline PANSS_1–7 _Total score at Week 8. Time to loss of response was significantly longer with olanzapine when compared to risperidone (p < .001), quetiapine (p = .003), or ziprasidone (p = .008 and p = .03), but not when compared to aripiprazole (p = .97). To provide clinical context, a table beneath each KM curve provides, by treatment group, the day at which >10% and >25% of patients who had initially responded lost response. All times were estimable at the >10% loss level, and at this level, olanzapine prolonged response by almost 10 weeks versus risperidone, by over 7 weeks versus quetiapine, by 3–4 weeks versus ziprasidone, and by 4 weeks versus aripiprazole.

A sensitivity analysis using a different scoring system for the PANSS and different thresholds for response and loss of response revealed similar results. Time to loss of response was statistically longer with olanzapine compared to risperidone (p < .001) and quetiapine (p = .003). Though time to loss of response was longer with olanzapine than with ziprasidone, this difference no longer reached statistical significance in the sensitivity analysis (p = .09 and p = .20).

The proportion of patients who lost response following Week 8 is shown by treatment group for each study in Table 2. For patients who achieved response, those treated with olanzapine had a significantly lower rate of loss of response after Week 8 than those treated with risperidone, quetiapine, and ziprasidone. Patients in the risperidone, quetiapine, and ziprasidone groups were 2.5, 3.2, 1.7, and 4.2 times more likely, respectively, to lose response than patients treated with olanzapine. The NNT with olanzapine rather than comparator to avoid loss of one additional responder over 24 or 28 weeks of treatment is shown by study in Table 2. NNTs were low with a range of 5 to 9, favoring olanzapine against all comparators except aripiprazole.

**Table 2:** Proportion of patients shown by treatment group who lost response after Week 8*.

**Study**	**Treatment**	**Patients who Lost** **Response, % (n/N)**	**p value^a^**	**NNT** **(95% CI)^b^**
Tran [13]	Olanzapine	11.4% (12/105)	.002	
	Risperidone	28.7% (27/94)		6 (4, 16)

Kinon [14]	Olanzapine	9.6% (5/52)	.01	
	Quetiapine	31.1% (14/45)		5 (3, 18)

Breier [17]	Olanzapine	16.9% (28/166)	.02	
	Ziprasidone	29.3% (36/123)		9 (5, 40)

Kinon [16]	Olanzapine	4.9% (3/61)	.02	
	Ziprasidone	20.4% (10/49)		7 (4, 34)

Kane [15]	Olanzapine	13.1% (20/153)	.999	
	Aripiprazole	12.5% (17/136)		-175 (-13, 15)

Abbreviations: PANSS = Positive and Negative Syndrome Scale; CGI-S = Clinical Global Impression Severity Index; n = number of patients who lost response after Week 8; N = number of patients who had response at Week 8; NNT = number needed to treat; CI = confidence interval.

^a ^Fisher's exact test.

^b ^NNT = 1/Absolute Risk Reduction, with 95% CI calculated as previously described [22].

* Proportion of patients who lost response (≥ 20% worsening of PANSS Total score and CGI-S score ≥ 3 occurring any time after Week 8) after having achieved response (≥ 20% improvement over baseline PANSS Total score at Week 8) for 5 randomized, double-blind studies of olanzapine versus another atypical comparator. Also shown are the numbers needed to treat (NNT) with olanzapine rather than comparator to avoid loss of one additional responder.

In one of the two studies in which ziprasidone was the comparator, patients treated with olanzapine spent a higher proportion of study time in response (63.0% versus 50.6% [p = .002]). There were no significant differences in this measure between olanzapine and the risperidone, quetiapine, and aripiprazole groups.

## Discussion

In this post-hoc analysis of 5 randomized, double-blind trials of olanzapine versus other atypical antipsychotics, patients treated with olanzapine who responded at Week 8 maintained their treatment response longer than did patients treated with quetiapine, risperidone, or ziprasidone. Also, in one of two studies, patients treated with olanzapine spent a greater percentage of cumulative days in response following randomization than did patients treated with ziprasidone. The low NNTs associated with these differences mean that relatively few patients would need to be treated with olanzapine compared to risperidone, quetiapine, or ziprasidone to prevent 1 additional loss of response in patients who initially achieved response.

Poor adherence to antipsychotic therapy is a clinically significant issue in the care of patients with schizophrenia and has notable impact on long-term disease outcome [1,2], resource utilization [23], and quality of life [2]. Recent data suggest that a major reason for medication discontinuation is lack of initial efficacy [4] and later, loss of efficacy [5]. Efficacy is clearly important, but researchers and clinicians are uncertain as to how to accurately measure this complex and multidimensional concept. Increasingly, efficacy measurements have incorporated clinically meaningful categorical definitions and time elements that reflect appreciation of schizophrenia as a chronic illness with episodes of response, prolonged response, remission, relapse, and recovery [24]. In this analysis, we have provided comparative efficacy data for 4 atypical antipsychotic agents using an alternative measure of efficacy; duration of time until loss of response.

To assess efficacy, we used scale-derived cut-offs that had clinical relevance, as had been suggested by Leucht et al. [11]. For the nearly 50% of patients in our study with baseline PANSS Total scores at or near 90, a 20% improvement in score signified clinical improvement from "severely ill" to "moderately ill". Through use of sensitivity analyses, we again followed recommendations by Leucht et al. [9] to provide results using more than one cut-off point and to score PANSS items from 0–6. Results of our sensitivity analysis were consistent with the primary analyses, suggesting our data were robust

In the CATIE schizophrenia study, researchers used a measure of efficacy that included a time element; the number of months in successful treatment, where successful treatment was defined as having a CGI score ≤ 3 (mildly ill) or a score of 4 (moderately ill) with an improvement of at least 2 points from baseline. The duration of successful treatment was significantly longer for patients treated with olanzapine compared to quetiapine, risperidone, and perphenazine treatment, and for patients treated with risperidone compared to those treated with quetiapine [3]. We have, in part, replicated this efficacy ranking, and extended the assessment by adding an additional symptom-based measure of response, the PANSS Total score.

Our results also replicated those of Sethuraman et al. [25], who found that during 28 weeks of observation, olanzapine-treated patients spent more cumulative time in remission than risperidone-treated patients. This finding held true whether remission was defined by the criteria of an expert consensus panel [26] or by criteria used in a study of treatment-naive patients treated for 52 weeks [27]. In a similar manner, olanzapine has proven superior to risperidone and quetiapine in large, randomized clinical trials measuring time to discontinuation for any cause [3,28], echoing our results and suggesting that antipsychotic adherence is often driven by efficacy.

No significant difference was found between treatment with olanzapine and aripiprazole in time to loss of response for patients who met criteria for response at Week 8. However, in the aripiprazole source study used here, a 28-week Lilly-sponsored randomized, double-blind trial, and in a 52-week study sponsored by Bristol-Myers Squibb [29] (BMS), olanzapine was superior to aripiprazole in mean change from baseline in PANSS Total score beginning at Week 6 and extending through Week 52. In addition, while discontinuation rates at 6 months were not different between groups in the Lilly-sponsored study, olanzapine-treated patients in the BMS-sponsored study had lower rates of discontinuation throughout the study. Olanzapine-treated patients had significantly more weight gain and triglyceride elevations in both studies. Data regarding time maintaining response for the 52-week BMS study have not been published.

This analysis has several limitations. First, given that there is no established definition for loss of response, we have created multiple definitions (cut-off percentages for improvement and worsening of PANSS scores, an absolute cut-off for CGI score, and a time limit for response) based on previous studies and treatment guidelines. It could be argued that our results would have been different had different parameters been chosen. For example, in defining response as a change in PANSS Total score at Week 8, we failed to include those patients who met criteria for response prior to Week 8, but who were unable to sustain it. A recent analysis by Kinon et al. showed that there exists a well-defined subset of patients who demonstrate minimal to moderate clinical improvement by Week 2, then worsen (and often discontinue treatment) between Weeks 5 and 8 [5]. These responders have not been represented in this analysis

*A priori*, we chose to use strict cut-offs of 196 days for 28-week studies and 168 days for 24-week studies, omitting any data gathered subsequent to these limits. A weakness inherent in evaluating survival plots is that conclusions based on data from the far right of the figure come from fewer patients and are therefore less certain. In these analyses, strict time cut-offs were employed to minimize this issue and to keep consistent with source study protocols.

Although large numbers of patients entered these trials, only 54% of patients randomly allocated to receive olanzapine and 44% of those allocated to other antipsychotic medication completed treatment through Week 24 or Week 28. This low completion rate is in keeping with other long-term studies of antipsychotic adherence, but limits the strength and generalizability of our results. Whether the cohort of patients who discontinued the study would have provided similar results is not known.

In 2005, the Remission in Schizophrenia Working Group published a definition of remission in which specific response criteria had to be maintained for ≥ 6 months [26]. This study offers little to advance understanding of remission. Though we had the necessary response data to assess remission, we were limited by inadequate study lengths and high drop-out rates.

Finally, these analyses do not address safety issues, another important factor in choosing an antipsychotic. However, much has been written about the safety issues associated with each of the medications included in this analysis. In particular, we note that olanzapine has the potential for significant weight gain in more than one-fourth of patients during short-term use and in more than one-half of patients during long-term use [30]. Ultimately, decisions regarding antipsychotic choice must be made after careful consideration of a medication's benefit and risk, in light of individual patient vulnerabilities.

## Conclusion

In this study, we have provided data on how treatment with olanzapine compares to treatment with other antipsychotics in maintaining treatment response. Maintenance of response is an outcome measure that adds depth and dimension to our understanding of efficacy in the treatment of schizophrenia. The source studies for these analyses were double-blind, randomized trials of significant duration, and included a large, relatively ill, yet diverse patient population. We found that olanzapine was superior to quetiapine, risperidone, and ziprasidone in maintaining response once patients had achieved response. There was no significant difference between treatment with olanzapine and aripiprazole. The robustness of these results was reinforced by sensitivity analyses.

## Competing interests

The studies included in this analysis were sponsored by Eli Lilly and Company, the manufacturer of olanzapine. VS, HAS, LL, and RC are all employees of Eli Lilly and Company. TB is a scientific writer employed full-time by i3 Statprobe, a division of Ingenix, which is a subsidiary of UnitedHealth Group. Eli Lilly contracted with i3 Statprobe for assistance with this manuscript.

## Authors' contributions

VS, HAS, LL, and RC conceived of the study and participated in the design of the study. VS, HAS, and LL acquired the data; LL performed the data analysis. VS, HAS, LL, TB, and RC participated in the interpretation of the data. TB drafted the manuscript. All authors were involved in critical revision of this manuscript, and provided final approval prior to submission.

## Pre-publication history

The pre-publication history for this paper can be accessed here:


